# (1-C5H14N2Br)_2_MnBr_*4*_: A Lead-Free Zero-Dimensional Organic-Metal Halide With Intense Green Photoluminescence

**DOI:** 10.3389/fchem.2020.00352

**Published:** 2020-04-28

**Authors:** Xiaomei Jiang, Zhaolai Chen, Xutang Tao

**Affiliations:** State Key Laboratory of Crystal Materials & Institute of Crystal Materials, Shandong University, Jinan, China

**Keywords:** organic-metal halides, photoluminescence, single crystal, lead-free materials, manganese

## Abstract

Low-dimensional organic-inorganic hybrid materials have attracted tremendous attentions due to their fascinating properties as emerging star materials for light-emitting applications. Taking advantage of their rich chemical composition and structural diversity, here, a novel lead-free organic-manganese halide compound, (1-mPQBr)_2_MnBr_4_ (1-mPQ = 1-methylpiperazine, 1-C5H14N2) with zero-dimensional structure has been rationally designed and successfully synthesized through solvent-evaporation method. Systematical characterizations were carried out to investigate the structure, thermal and photophysical properties. The (1-mPQBr)_2_MnBr_4_ was found to crystallized into an orthorhombic crystal (P2_1_2_1_2_1_) with lattice parameters of a = 8.272(6) Å, b = 15.982(10) Å and c = 17.489(11) Å. The structure consists of isolated [MnBr_4_]^2−^ clusters and free Br^−^ ions as well as [C5H14N2]^2+^ molecules. Thermal analysis indicates that it is stable up to 300°C. Upon ultraviolet photoexcitation, the (1-mPQBr)_2_MnBr_4_ exhibits intense green emission centered at 520 nm with a narrow full width at half-maximum of 43 nm at room temperature, which should be assigned to the spin-forbidden internal transition (^4^T_1_(G) to ^6^A_1_) of tetrahedrally coordinated Mn^2+^ ions. The superior photoluminescence properties coupled with facile and efficient synthesis method of this material suggest its considerable promise to be utilized as light-emitting materials.

## Introduction

In recent decades, organic metal halide materials have flourished as star materials in solution-processed optoelectronics fields (Kojima et al., 2009; Chen et al., 2017; Yang et al., 2019a), arising from their superior properties including high absorption coefficient, long electron-hole diffusion length, ultralow trap density and high photoluminescence quantum yield (PLQY) as well as facile synthesis including low cost, high efficiency and flexibility (Dang et al., 2015; Liu et al., 2015, 2018; Huang et al., 2017). Benefitting from their remarkable advantages, they have shown great potential for photovoltaic solar cells (Kojima et al., 2009; Cheng et al., 2019; Yang et al., 2019b), light-emitting diodes (Ling et al., 2016; Thirumal et al., 2017; Lin et al., 2018), photodetectors (Adinolfi et al., 2016; Ahmadi et al., 2017; Shrestha et al., 2017), field-effect transistors (Yu et al., 2018; Zhu et al., 2019), and lasers (Yakunin et al., 2015; Zhu et al., 2015; Gu et al., 2016). Lately, the certified power conversion efficiency of organic lead halide solar cells have achieved 25.2%^1^, which outperforms that of CdTe and CuInGaSe_2_ solar cells. In spite of their rapid development, the presence of toxic lead in these materials is deemed to be a seriously concern, becoming a huge hindrance in their way to wide-scale exploitation. Based on this circumstance, it is therefore of urgent need to look for the alternative lead-free hybrids for future commercial optoelectronic applications.

Hence, immense efforts in reducing lead contents or exploring lead-free substitutes offer a viable solution for high-performance eco-friendly optoelectronic devices. Noteworthy, this class of materials endows with rich chemical and structural diversities. In addition to modifying the length of the organic components, the diverse structural dimensionality, referring to three-dimensional (3D), two-dimensional (2D), one-dimensional (1D) and zero-dimensional (0D) structures, can also be achieved by tuning the inorganic frameworks, which results in fascinating properties. In the first place, the most obvious alternative substitution to Pb^2+^, should be the elements in the same group in the periodic table, namely Sn^2+^ and Ge^2+^ (Zhumekenov et al., 2017; Fu et al., 2018; Ju et al., 2018a; Nazarenko et al., 2019). In addition, heterovalent elements in Group 15 (Bi^3+^ and Sb^3+^) (Abulikemu et al., 2016; Sun et al., 2016; Ji et al., 2017, 2018; Ju et al., 2018b; Zhang et al., 2018; Tao et al., 2019) and in Group 13 (In^3+^) (Zhou et al., 2019) have also been demonstrated as the alternative metals. Moreover, a range of divalent transition metal ions [Cu^2+^ (Cortecchia et al., 2016; Jun et al., 2018; Li et al., 2018; Park et al., 2018), Fe^2+^ (Han et al., 2014, 2015; Nakayama et al., 2017), Mn^2+^ (Bai et al., 2018; Park et al., 2018)] can also serve as substitutes for Pb^2+^. Among which, large number of researches have reported that organic manganese (Mn^2+^) halides possess brilliant photoluminescence ranging from green to red due to the variable metal-ion coordination geometry, with the photoluminescence lifetimes varying from microseconds to milliseconds. Many groups have made great efforts in exploring octahedral- coordinated Mn (Han et al., 2015; Lv et al., 2016; Nakayama et al., 2017; Bai et al., 2018) single crystals, such as 3D-structured (CH_3_)_3_NCH_2_ClMnCl_3_ (You et al., 2017) and (3-Pyrrolinium)MnX_3_ (X = Cl, Br) (Ye et al., 2015), 2D-structured NH_3_(CH_2_)_5_NH_3_MnCl_4_ (You et al., 2017) and (C_6_H_5_CH_2_CH_2_NH_3_)_2_MnCl_4_ (Lv et al., 2016), 1D-structured (N-Methylpyrrolidinium)MnCl_3_ (Ye et al., 2015), and (pyrrolidinium)MnBr_3_ (Sun et al., 2017), as well as 0D-structured (C4NOH10)_5_Mn_2_Cl_9_·C2H5OH (Zhang et al., 2015), etc. In comparison, less attention has been paid on the search and investigation of tetracoordinated Mn^2+^ counterparts (Xu et al., 2017; Gong et al., 2019; Jana et al., 2019; Sun et al., 2019).

It is well-known that single crystals can exhibit better intrinsic properties of materials compared with the polycrystalline counterparts. Hence, in this work, we first rationally designed and synthesized a novel organic manganese halide, (1-mPQBr)_2_MnBr_4_ (1-mPQ=1-methylpiperazine, 1-C5H14N2) single crystal with a 0D structure. Systematical characterizations were applied to investigate the structures, photophysical and thermal properties. The facile solvent-evaporation method, the intense green emission with high PLQY of 60.70% as well as good stability makes the (1-mPQBr)_2_MnBr_4_ suitable as green phosphors.

## Materials and Methods

### Materials

Analytical-grade manganese (II) monoxide (MnO, 99.0%, Sinopharm Co. Ltd.), 1-methylpiperazine (1-C5H12N2, 99.0%, Sinopharm Co. Ltd.), hydrobromic acid (HBr, 40 wt% in H_2_O, Sinopharm Co. Ltd.), and hypophosphorous acid aqueous solution (H_3_PO_2_, 50% in H_2_O, Sinopharm Co. Ltd.) were used as received without any further processing or refining.

### Preparation of (1-mPQBr)_2_MnBr_4_ Single Crystal

As a typical process, the yellow crystals of (1-mPQBr)_2_MnBr_4_ were obtained by slowly evaporating HBr/H_3_PO_2_ (3:1) mixed solutions containing 1-methylpiperazine and MnO with molar amounts of 2:1.

### Characterizations

**Powder X-ray diffraction (PXRD) patterns** were performed on a Bruker-AXS D8 Advance X-ray diffractometer with CuKα1 radiation (λ = 1.54186 Å) in the range of 10–90° (2θ).

**Single crystal's structure** was determined by Bruker SMART APEX-II diffractometer equipped with a CCD detector (graphite-monochromatized Mo-Kα radiation, λ = 0.71073 Å) at 300 K. Data integration and cell refinement were performed using the APEX_2_ software. The structure was analyzed by direct methods and refined using the SHELXTL 97 software package. All non-hydrogen atoms of the structure were refined with anisotropic thermal parameters, and the refinements converged for Fo^2^ > 2σIJFo^2^. All the calculations were performed using SHELXTL crystallographic software package. Symmetry analysis on the model using PLATON revealed that no obvious space group change was needed. The crystallographic data was deposited in Cambridge Crystallographic Data Center (CCDC #1979443).

**Fourier transform infrared (FTIR)** spectrum in the region 700–4,000 cm^−1^ was examined on a spectrometer (Nicolet 330) with KBr pellets.

**X-ray photoelectron spectroscopy (XPS) measurements** of the newly synthesized (1-mPQBr)_2_MnBr_4_ samples about 2 × 1 × 1 mm^3^ in size were performed on an ESCALAB 250 (Thermo Fisher Scientific) instrument under vacuum (1.7 × 10^−10^ mbar).

**UV-vis diffuse reflectance spectroscopy** was recorded using a Shimadzu UV 2550 spectrophotometer equipped with an integrating sphere over the spectral range 200–800 nm. The (1-mPQBr)_2_MnBr_4_ single crystals were ground into powders for tests. A BaSO_4_ plate was used as the standard (100% reflectance). The absorption spectrum was calculated from the reflectance spectrum by using the Kubelka-Munk function: α/S = (1–R)^2^/(2R), where α is the absorption coefficient, S is the scattering coefficient, and R is the reflectance.

### Photoluminescence Measurements

The excitation wavelength dependent-photoluminescence (PL) spectra and PL excitation spectra (PLE) were carried out by a laser of 365 nm with a photomultiplier (PMTH-S1-CR131) and DSP lock-in amplifier (SRS 830). The time-resolved photoluminescence measurements (TRPL) were carried by FLS920 all functional fluorescence spectrometer (Edinburgh). The output laser wavelength was set to be 520 nm. The photoluminescence quantum yields (PLQY) were tested by an absolute PLQY measurement system (FLSP920) in an integrating sphere.

### Thermalgravimetric Analysis (TGA) and Differential Scanning Calorimetry (DSC) Measurements

TGA and DSC curves were collected using a TGA/DSC1/1600HT analyzer (Metter Toledo Instruments). The polycrystalline sample was placed in an aluminum crucible and heated at a rate of 10 K/min from room temperature to 800°C under flowing nitrogen gas.

## Results and Discussion

**Figure 1:**
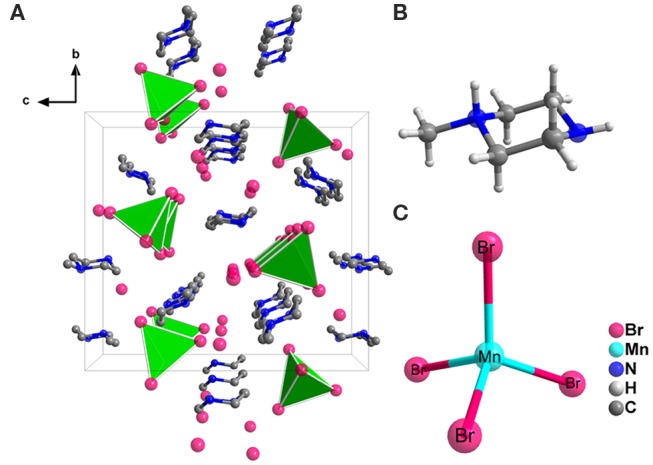
**(A)** Single crystal structural packing of (1-mPQBr)_2_MnBr_4_ viewed along a-axis direction (H atoms are omitted for clarity). Ball-and-stick scheme of **(B)** a single [C5H14N2]^2+^ organic cation and **(C)** a single [MnBr_4_]^2−^ tetrahedron.

**Figure 2:**
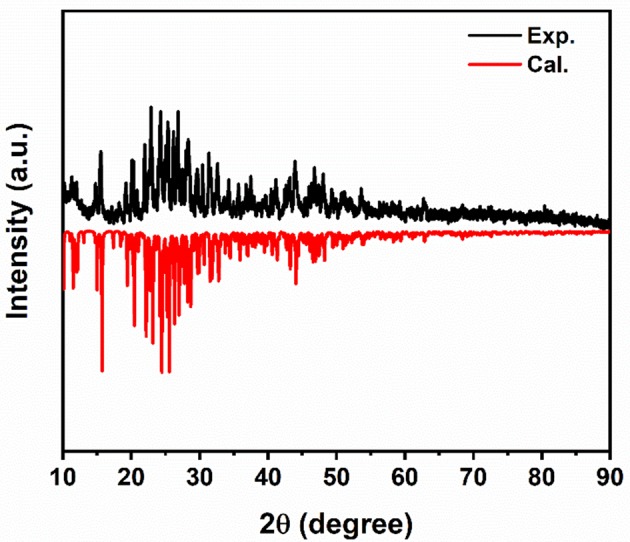
Simulated SCXRD and experimental PXRD patterns of (1-mPQBr)_2_MnBr_4_ powders at 300 K.

Crystal structure of the (1-mPQBr)_2_MnBr_4_ single crystal was obtained through SCXRD test, which belongs to the orthorhombic crystal system (a non-polar D_2_ and chiral space group P2_1_2_1_2_1_) at room temperature, with lattice parameters of a = 8.272(6) Å, b = 15.982(10) Å and c = 17.489(11) Å. In this structure, four formula units of (1-mPQBr)_2_MnBr_4_ are present in the unit cell. Further details for crystallographic parameters are provided in Tables S1–S3 (in the Supporting Information) and the crystal structure and local structure descriptions are displayed in Figure 1. It is clearly seen that each Mn atom is coordinated by four Br atoms to form an isolated [MnBr_4_]^2−^ tetrahedral cluster as inorganic part of the title compound. Such isolated [MnBr_4_]^2−^ tetrahedral are surrounded by free Br^−^ and [C5H14N2]^2+^ molecules (organic part), featuring a 0D structure. In Figure 2 shows that the experimental PXRD pattern corresponds well to that calculated from SCXRD result with slightly varying intensities.

The FTIR spectrum in Figure S1 further verifies the existence of the organic component. The broad peak around 3,353 cm^−1^ belongs to the N-H stretching peak. The peaks in the range of 2,950–2,820 cm^−1^ are assigned to the CH2 and CH3 symmetric and asymmetric stretching vibrations and the peak around 1,410 cm^−1^ is ascribed to CH bending vibrations. Also, the strong signal at nearly 1,630 cm^−1^ indicates asymmetric ${\text{NH}}_{3}^{+}$ deformation. Figure S2 shows the scanning electron microscope photograph of (1-mPQBr)_2_MnBr_4_ crystal. Table S4 provides the detailed results of energy dispersive X-ray spectroscopy to further confirm the composition of inorganic and organic parts, respectively.

**Figure 3:**
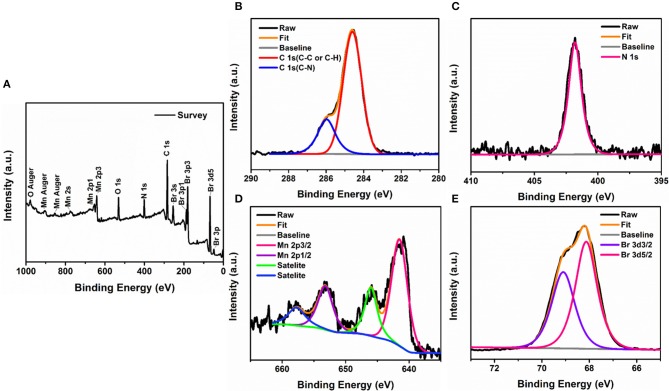
**(A)** XPS survey spectra of (1-mPQBr)_2_MnBr_4_. High-resolution XPS spectra of **(B)** C 1s, **(C)** N 1s, **(D)** Mn 2p, and **(E)** Br 3d.

Additionally, the XPS spectrum in Figure 3A evidences the signatures of carbon (C 1s), oxygen (O 1s), nitrogen (N 1s), manganese(Mn 2p), and bromide (Br 3d), and the appearance of adventitious oxygen in the spectrum is generally ascribed to a contamination of the sample by physical adsorption during ambient exposure. We further recorded high-resolution spectra of the constituents in designated energy ranges (Figures 3B–E). The Mn doublet shows a spin-orbit splitting of 11.7 eV with the peaks corresponding to the binding energies of Mn 2p1/2 and 2p3/2 orbitals located at 653.3 and 641.6 eV, respectively. Similarly, the core-level spectrum of Br 3d contains a couple of split peaks at 69.2 and 68.1 eV corresponding to Br 3d3/2 and Br 3d5/2 orbitals with a separation of 1.1 eV, which are in good agreement with Br. By calculating the integrated peak areas of the XPS spectra, we can roughly estimate that Mn to Br possessed a molar ratio of 1:5.88. According to the analytical results of SCXRD, PXRD, EDS, and XPS, we can verify that pure (1-mPQBr)_2_MnBr_4_ were successfully synthesized through the solvent-evaporation method.

**Figure 4:**
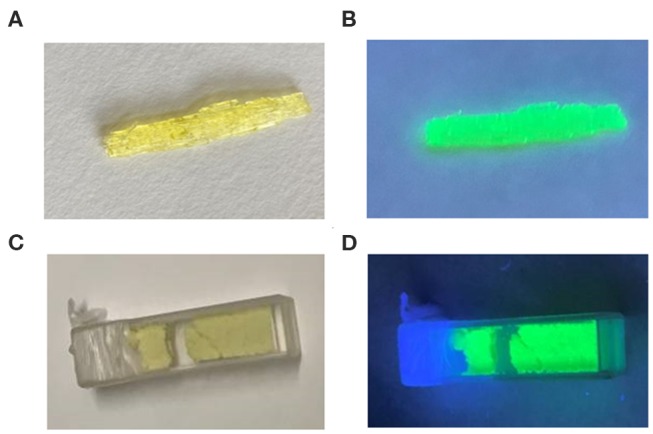
Optical photograph of (1-mPQBr)_2_MnBr_4_ single crystal and powders **(A,B)** without UV light excitation, **(C,D)** with 365 nm UV light excitation.

**Figure 5:**
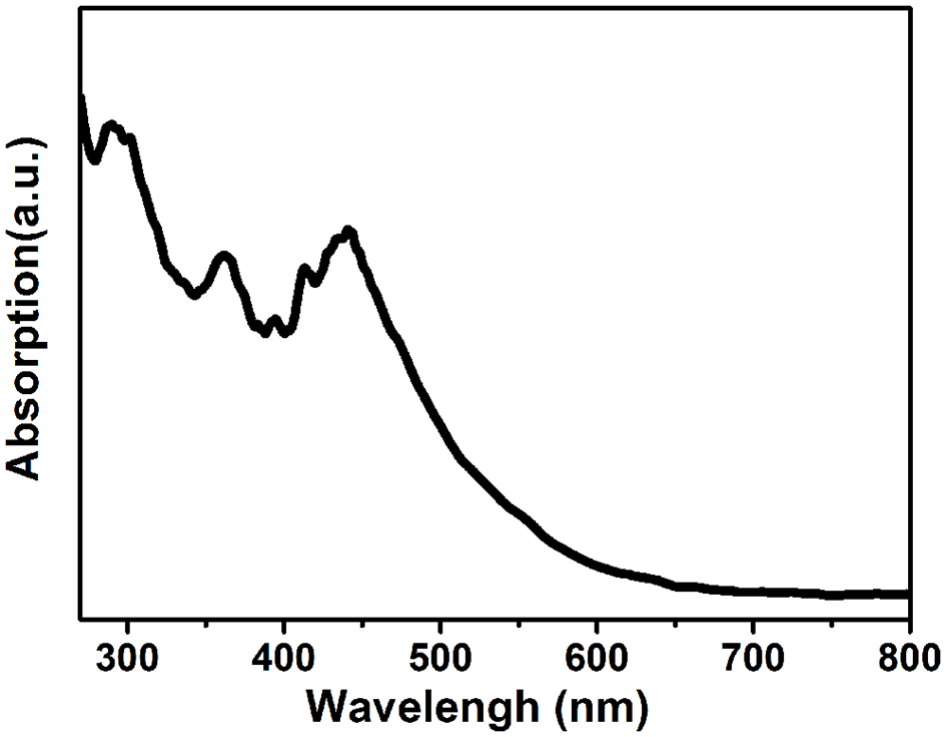
UV-visible absorption spectrum of (1-mPQBr)_2_MnBr_4_.

Figure 4 displays the optical image of a rod-shaped (1-mPQBr)_2_MnBr_4_ single crystal and powders under ultraviolet light irradiation, and it is clearly seen that the title compound emits strong green light. To characterize the photophysical properties of (1-mPQBr)_2_MnBr_4_, the UV-vis absorption spectroscopy and PL spectroscopy were carried out at room temperature. In Figure 5, typical absorption spectra for organic manganese halides materials can be observed. The peaks below 340 nm originates from the transitions within the [C5H14N2]^2+^ cation and peaks from 345 to 600 nm should be ascribed to the electronic transitions between the ground and the first excited triplet states of the Mn^2+^ ion in the crystal field, which is consistent with previous reports as listed in Table S5.

**Figure 6:**
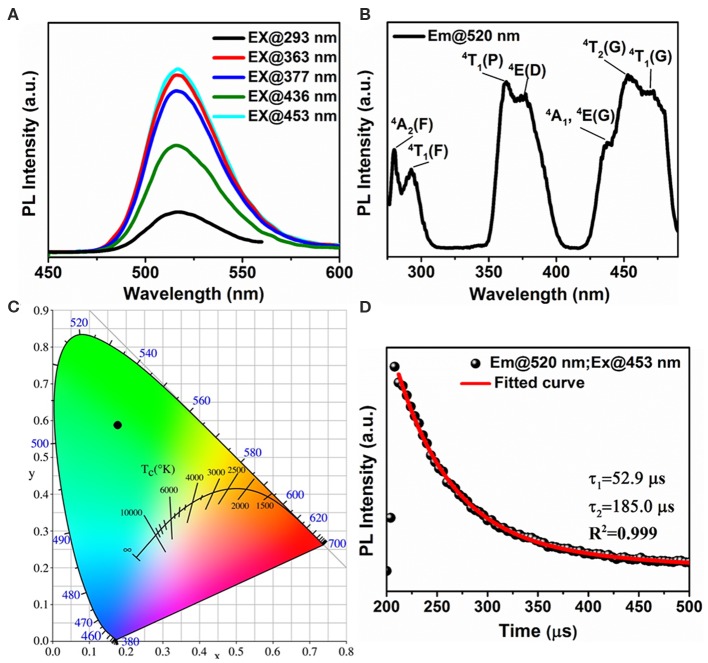
Optical properties of (1-mPQBr)_2_MnBr_4_: **(A,B)** wavelength-dependent PL spectra and PLE spetrum at room temperature, **(C)** CIE chromaticity coordinates, **(D)** TRPL decay curve monitored at 520 nm under 453 nm excitation.

To further investigate the origin of this green emission, Figures 6A,B display the wavelength dependent PL spectra and PLE spectrum of (1-mPQBr)_2_MnBr_4_ single crystals. Upon excitation, the (1-mPQBr)_2_MnBr_4_ exhibits an intense green emission located at 520 nm with a narrow full width at half-maximum (FWHM) of 43 nm, corresponding to a characteristic transition from the ground state of the d-electron configuration (e_g_)^2^ (t_2g_)^3^ to the upper state of the configuration (e_g_)^3^ (t_2g_)^2^ (Wrighton and Ginley, 1974; Jiang et al., 2019). It is clearly noted that the PLE spectra are consistent with the absorption spectra. In the region between 300 and 500 nm, the discernable peaks correspond to radiative transitions from the ground state ^6^A_1_ of tetrahedral Mn (II) to the excited states of ^4^T_1_(G), ^4^T_2_(4G), ^4^A_1_(G), ^4^E(G), 4E(D), ^4^T_1_(P), ^4^T_1_(F), and ^4^A_2_(F), respectively, according to the excited states of Mn^2+^ system (d5) in the Tanabe-Sugano diagram (Rodríguez-Lazcano et al., 2009).

The PLQY at room temperature is calculated to be about 60.70% and the Commission Internationale de l'Eclairage (CIE) chromaticity coordinate for this green emission is determined to be (0.175, 0.589) (Figure 6C). Upon the excitation of 453 nm, the room-temperature TRPL decay curve is monitored for the emission peak at 520 nm as shown in Figure 6D. The decay curve is modeled with the biexponential decay function:

$$\begin{array}{l} I\left(t\right)=A_{1}e^{-\frac{t}{\tau_{1}}}+A_{2}e^{-\frac{t}{\tau_{2}}} \end{array}$$

where I(*t*) is the time-resolved PL intensity, t is the time after excitation, A_1_ and A_2_ are the relative amplitudes, and τ_1_ and τ_2_ are lifetimes for fast and slow decays. The effective decay times are calculated to be 52.9 and 185.0 μs, respectively.

**Figure 7:**
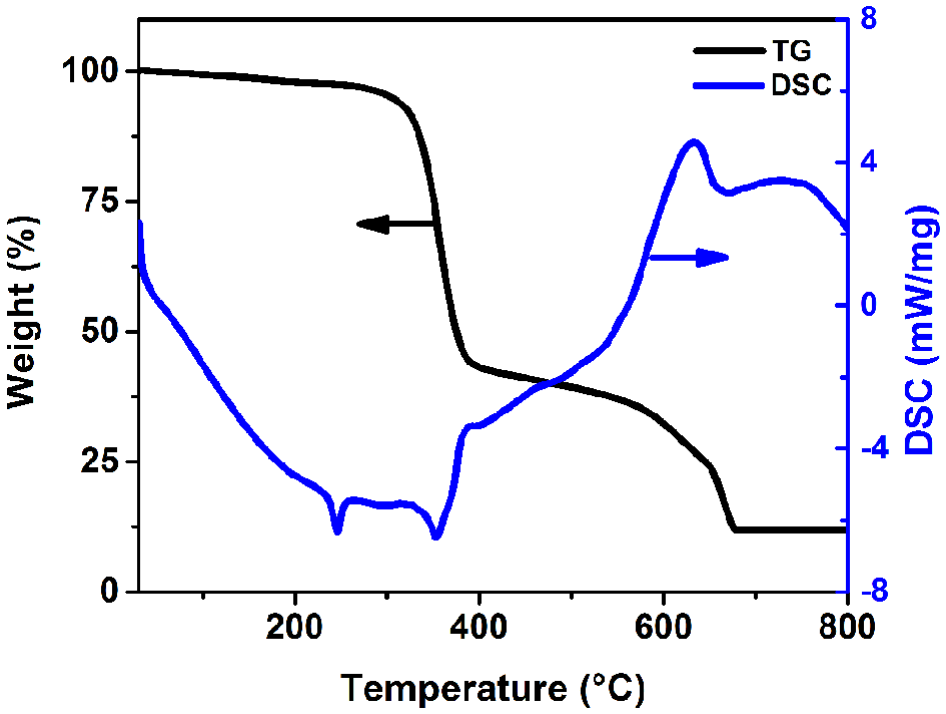
TGA and DSC data for (1-mPQBr)_2_MnBr_4_.

Furthermore, such highly emissive bulk crystals and powders were examined to exhibit considerable thermal stability. TGA curve suggests that (1-mPQBr)_2_MnBr_4_ does not lose any mass until 300°C (Figure 7), which is comparatively higher than that of other organic-manganese halides reported in literature. This is hypothesized to be due to the large amounts of hydrogen bonding interaction between the organic and inorganic components in the 0D structure benefiting from the extra presence of free bromide ions. It possesses a two-step decomposition including the evaporation of organic parts and MnBr_2_, respectively. In DSC scan, a sharp endothermic peak, which occurred at 250°C, corresponds to the melting point of (1-mPQBr)_2_MnBr_4_.

More importantly, the stability of hybrid metal halides is deemed as an important criterion for evaluation of their potential for practical applications. Therefore, we evaluated the thermal stability of (1-mPQBr)_2_MnBr_4_ single crystals by annealing them at 150°C for 12 h on a hotplate. Notably, negligible change can be observed in the PXRD pattern (Figure S3). Moreover, after exposure to ambient conditions for 2 months, it still remain 96.3% of the original PL intensity (Figure S4).

## Conclusions

In summary, we have synthesized a novel lead-free organic-manganese halide compound (1-mPQBr)_2_MnBr_4_ (1-mPQ=1-methylpiperazine, 1- C5H14N2), with 0D structure through solvent-evaporation method. A highly luminescent green emission at 520 nm was observed for this novel organic-inorganic hybrid material, which should be resulted from the spin-forbidden internal transition (^4^T_1_(G) to ^6^A_1_) of tetrahedrally coordinated Mn^2+^ ions. We believe the superior photophysical properties and high stability makes it potential for light-emitting applications.

## Data Availability Statement

The datasets presented in this study can be found in online repositories. The name of the repository and accession number can be found below: Cambridge Crystallographic Data Centre (CCDC #1979443).

## Author Contributions

ZC and XT devised the project and proof outline. XJ synthesized the single crystals and conducted all the characterizations. All authors contributed to manuscript revision, read, and approved the submitted version.

## Conflict of Interest

The authors declare that the research was conducted in the absence of any commercial or financial relationships that could be construed as a potential conflict of interest. The reviewer JD declared a past co-authorship with the authors XJ, ZC, and XT to the handling editor.

## Footnotes

^1^National Renewable Energy Labs (NREL) Efficiency Chart. Available online at: https://www.nrel.gov/pv/assets/pdfs/best-research-cell-efficiencies.20200203.pdf.

## References

[B1] Abulikemu M. Ould-Chikh S. Miao X. Alarousu E. Murali B. Ngongang Ndjawa G. O. (2016). Optoelectronic and photovoltaic properties of the air-stable organohalide semiconductor (CH_3_NH_3_)_3_Bi_2_I_9_. J. Mater. Chem. A. 4, 12504–12515. 10.1039/C6TA04657F

[B2] Adinolfi V. Ouellette O. Saidaminov M. I. Walters G. Abdelhady A. L. Bakr O. M.. (2016). Fast and sensitive solution-processed visible-blind perovskite UV photodetectors. Adv. Mater. 28, 7264–7268. 10.1002/adma.201601196 27300753

[B3] Ahmadi M. Wu T. Hu B. (2017). A review on organic-inorganic halide perovskite photodetectors: device engineering and fundamental physics. Adv. Mater. 29:1605242. 10.1002/adma.201605242 28910505

[B4] Bai X. W. Zhong H. Z. Chen B. K. Chen C. Han J. B. Zeng R. S. (2018). Pyridine-modulated mn ion emission properties of C_10_H_12_N_2_MnBr_4_ and C_5_H_6_NMnBr_3_ Single crystals. J. Phys. Chem. C. 122, 3130–3137. 10.1021/acs.jpcc.7b11693

[B5] Chen Z. Dong Q. Liu Y. Bao C. Fang Y. Lin Y.. (2017). Thin single crystal perovskite solar cells to harvest below-bandgap light absorption. Nat. Commun. 8:1890. 10.1038/s41467-017-02039-5 29192232 PMC5709415

[B6] Cheng X. Yang S. Cao B. Tao X. Chen Z. (2019). Single crystal perovskite solar cells: development and perspectives. Adv. Func. Mater. 30:1905021 10.1002/adfm.201905021

[B7] Cortecchia D. Dewi H. A. Yin J. Bruno A. Chen S. Baikie T.. (2016). Lead-Free MA_2_CuCl_(x)_Br_(4−x)_ hybrid perovskites. Inorg. Chem. 55, 1044–1052. 10.1021/acs.inorgchem.5b01896 26756860

[B8] Dang Y. Liu Y. Sun Y. Yuan D. Liu X. Lu W. (2015). Bulk crystal growth of hybrid perovskite material CH_3_NH_3_PbI_3_. CrystEngComm. 17, 665–670. 10.1039/C4CE02106A

[B9] Fu P. Huang M. Shang Y. Yu N. Zhou H. L. Zhang Y. B.. (2018). Organic-inorganic layered and hollow tin bromide perovskite with tunable broadband emission. ACS Appl. Mater. Interfaces 10, 34363–34369. 10.1021/acsami.8b07673 30192511

[B10] Gong L. K. Hu Q. Q. Huang F. Q. Zhang Z. Z. Shen N. N. Hu B.. (2019). Efficient modulation of photoluminescence by hydrogen bonding interactions between inorganic [MnBr_4_]^2−^ anions and organic cations. Chem. Commun. 55, 7303–7306. 10.1039/C9CC03038G 31155621

[B11] Gu Z. Wang K. Sun W. Li J. Liu S. Song Q. (2016). Two-Photon Pumped CH3NH3PbBr3Perovskite microwire lasers. Adv. Opt. Mater. 4, 472–479. 10.1002/adom.201500597

[B12] Han J. Nishihara S. Inoue K. Kurmoo M. (2014). On the nature of the structural and magnetic phase transitions in the layered perovskite-like (CH_3_NH_3_)_2_[Fe(II)Cl_4_]. Inorg. Chem. 53, 2068–2075. 10.1021/ic402535u 24471961

[B13] Han J. Nishihara S. Inoue K. Kurmoo M. (2015). High magnetic hardness for the canted antiferromagnetic, ferroelectric, and ferroelastic layered perovskite-like (C_2_H_5_NH_3_)_2_[Fe(II)Cl_4_]. Inorg. Chem. 54, 2866–2874. 10.1021/ic5030229 25736878

[B14] Huang J. Yuan Y. Shao Y. Yan Y. (2017). Understanding the physical properties of hybrid perovskites for photovoltaic applications. Nat. Rev. Mater. 2:17042 10.1038/natrevmats.2017.42

[B15] Jana A. Zhumagali S. Ba Q. Nissimagoudar A. S. Kim K. S. (2019). Direct emission from quartet excited states triggered by upconversion phenomena in solid-phase synthesized fluorescent lead-free organic-inorganic hybrid compounds. J. Mater. Chem. A 7, 26504–26512. 10.1039/C9TA08268A

[B16] Ji C. Sun Z. Zeb A. Liu S. Zhang J. Hong M.. (2017). Bandgap narrowing of lead-free perovskite-type hybrids for visible-light-absorbing ferroelectric semiconductors. J. Phys. Chem. Lett. 8, 2012–2018. 10.1021/acs.jpclett.7b00673 28425290

[B17] Ji C. Wang P. Wu Z. Sun Z. Li L. Zhang J. (2018). Inch-size single crystal of a lead-free organic-inorganic hybrid perovskite for high-performance photodetector. Adv. Funct. Mater. 28:1705467 10.1002/adfm.201705467

[B18] Jiang X. Xia S. Zhang J. Ju D. Liu Y. Hu X. (2019). Exploring organic metal halides with reversible temperature-responsive dual-emissive photoluminescence. ChemSusChem 12, 5228–5232. 10.1002/cssc.201902481 31709721

[B19] Ju D. Dang Y. Zhu Z. Liu H. Chueh C.-C. Li X. (2018a). Tunable band gap and long carrier recombination lifetime of stable mixed CH_3_NH_3_Pb_x_Sn_1−x_Br_3_ single crystals. Chem. Mater. 30, 1556–1565. 10.1021/acs.chemmater.7b04565

[B20] Ju D. Jiang X. Xiao H. Chen X. Hu X. Tao X. (2018b). Narrow band gap and high mobility of lead-free perovskite single crystal Sn-doped MA3Sb2I9. J. Mater. Chem. A 6, 20753–20759. 10.1039/C8TA08315K

[B21] Jun T. Sim K. Iimura S. Sasase M. Kamioka H. Kim J. (2018). Lead-free highly efficient blue-emitting Cs_3_Cu_2_I_5_ with 0D electronic structure. Adv. Mater. 30:1804547 10.1002/adma.201804547 30216587

[B22] Kojima A. Teshima K. Shirai Y. Miyasaka T. (2009). Organometal halide perovskites as visible-light sensitizers for photovoltaic cells. J. Am. Chem. Soc. 131, 6050–6051. 10.1021/ja809598r 19366264

[B23] Li X. Li B. Chang J. Ding B. Zheng S. Wu Y. (2018). (C_6_H_5_CH_2_NH_3_)_2_CuBr_4_: a lead-free, highly stable two-dimensional perovskite for solar cell applications. ACS Appl. Energy Mater. 1, 2709–2716. 10.1021/acsaem.8b00372

[B24] Lin K. Xing J. Quan L. N. de Arquer F. P. G. Gong X. Lu J. (2018). Perovskite light-emitting diodes with external quantum efficiency exceeding 20 percent. Nature 562, 245–248. 10.1038/s41586-018-0575-3 30305741

[B25] Ling Y. Yuan Z. Tian Y. Wang X. Wang J. C. Xin Y.. (2016). Bright light-emitting diodes based on organometal halide perovskite nanoplatelets. Adv. Mater. 28, 305–311. 10.1002/adma.201503954 26572239

[B26] Liu Y. Yang Z. Cui D. Ren X. Sun J. Liu X. (2015). Two-Inch-Sized Perovskite CH_3_NH_3_PbX_3_ (X = Cl, Br, I) crystals: growth and characterization. Adv. Mater. 27, 5176–5183. 10.1002/adma.201502597 26247401

[B27] Liu Y. Zhang Y. Zhao K. Yang Z. Feng J. Zhang X. (2018). A 1300 mm^2^ ultrahigh-performance digital imaging assembly using high-quality perovskite single crystals. Adv. Mater. 30:e1707314 10.1002/adma.201707314 29845652

[B28] Lv X.-H. Liao W.-Q. Li P.-F. Wang Z.-X. Mao C.-Y. Zhang Y. (2016). Dielectric and photoluminescence properties of a layered perovskite-type organic-inorganic hybrid phase transition compound: NH_3_(CH_2_)_5_NH_3_MnCl_4_. J. Mater. Chem. C. 4, 1881–1885. 10.1039/C5TC04114G

[B29] Nakayama Y. Nishihara S. Inoue K. Suzuki T. Kurmoo M. (2017). Coupling of magnetic and elastic domains in the organic-inorganic layered perovskite-like (C_6_H_5_C_2_H_4_NH_3_)_2_Fe(II)Cl_4_ crystal. Angew. Chem. Int. Ed. Engl. 56, 9367–9370. 10.1002/anie.201703898 28621036

[B30] Nazarenko O. Kotyrba M. R. Yakunin S. Worle M. Benin B. M. Raino G.. (2019). Guanidinium and mixed cesium-guanidinium tin(II) bromides: effects of quantum confinement and out-of-plane octahedral tilting. Chem. Mater. 31, 2121–2129. 10.1021/acs.chemmater.9b00038 30930536 PMC6438322

[B31] Park G. Oh I. H. Park J. M. S. Jung J. You C. Y. Kim J. S.. (2018). Solvent-dependent self-assembly of two dimensional layered perovskite (C_6_H_5_CH_2_CH_2_NH_3_)_2_MCl_4_ (M = Cu, Mn) thin films in ambient humidity. Sci. Rep. 8:4661. 10.1038/s41598-018-23012-2 29549304 PMC5856791

[B32] Rodríguez-Lazcano Y. Nataf L. Rodríguez F. (2009). Pressure-induced transformation from isolated MnX4(Td) to exchange-coupled MnX_6_(Oh) in A_2_MnX_4_ (X: Cl, Br) crystals. Structural correlations by time-resolved spectroscopy. J. Lumin. 129, 2000–2003. 10.1016/j.jlumin.2009.04.077

[B33] Shrestha S. Fischer R. Matt G. J. Feldner P. Michel T. Osvet A. (2017). High-performance direct conversion X-ray detectors based on sintered hybrid lead triiodide perovskite wafers. Nat. Photonics. 11, 436–440. 10.1038/nphoton.2017.94

[B34] Sun M. E. Li Y. Dong X. Y. Zang S. Q. (2019). Thermoinduced structural-transformation and thermochromic luminescence in organic manganese chloride crystals. Chem. Sci. 10, 3836–3839. 10.1039/C8SC04711A 31015925 PMC6457203

[B35] Sun X. F. Li P. F. Liao W. Q. Wang Z. Gao J. Ye H. Y.. (2017). Notable broad dielectric relaxation and highly efficient red photoluminescence in a perovskite-type compound: (N-Methylpyrrolidinium)MnCl_3_. Inorg. Chem. 56, 12193–12198. 10.1021/acs.inorgchem.7b01553 28968076

[B36] Sun Z. Zeb A. Liu S. Ji C. Khan T. Li L.. (2016). Exploring a lead-free semiconducting hybrid ferroelectric with a zero-dimensional perovskite-like structure. Angew. Chem. Int. Ed. Engl. 55, 11854–11858. 10.1002/anie.201606079 27538754

[B37] Tao K. Li Y. Ji C. Liu X. Wu Z. Han S. (2019). A lead-free hybrid iodide with quantitative response to X-ray radiation. Chem. Mater. 31, 5927–5932. 10.1021/acs.chemmater.9b02263

[B38] Thirumal K. Chong W. K. Xie W. Ganguly R. Muduli S. K. Sherburne M. (2017). Morphology-independent stable white-light emission from self-assembled two-dimensional perovskites driven by strong exciton–phonon coupling to the organic framework. Chem. Mater. 29, 3947–3953. 10.1021/acs.chemmater.7b00073

[B39] Wrighton M. Ginley D. (1974). Excited state decay of tetrahalomanganese(ii) complexes. Chem. Phys. 4, 295–299. 10.1016/0301-0104(74)80097-2

[B40] Xu L. J. Sun C. Z. Xiao H. Wu Y. Chen Z. N. (2017). Green-light-emitting diodes based on tetrabromide manganese(II) complex through solution process. Adv. Mater. 29:1605739. 10.1002/adma.201605739 28009462

[B41] Yakunin S. Protesescu L. Krieg F. Bodnarchuk M. I. Nedelcu G. Humer M. (2015). Low-threshold amplified spontaneous emission and lasing from colloidal nanocrystals of caesium lead halide perovskites. Nat. Commun. 6:8056 10.1038/ncomms9056 26290056 PMC4560790

[B42] Yang S. Dai J. Yu Z. Shao Y. Zhou Y. Xiao X.. (2019b). Tailoring passivation molecular structures for extremely small open-circuit voltage loss in perovskite solar cells. J. Am. Chem. Soc. 141, 5781–5787. 10.1021/jacs.8b13091 30888171

[B43] Yang S. Xu Z. Xue S. Kandlakunta P. Cao L. Huang J. (2019a). Organohalide lead perovskites: more stable than glass under gamma-ray radiation. Adv. Mater. 31:e1805547. 10.1002/adma.201805547 30488496

[B44] Ye H. Y. Zhou Q. Niu X. Liao W. Q. Fu D. W. Zhang Y.. (2015). High-temperature ferroelectricity and photoluminescence in a hybrid organic-inorganic compound: (3-Pyrrolinium)MnCl_3_. J. Am. Chem. Soc. 137, 13148–13154. 10.1021/jacs.5b08290 26383504

[B45] You Y. M. Liao W. Q. Zhao D. Ye H. Y. Zhang Y. Zhou Q.. (2017). An organic-inorganic perovskite ferroelectric with large piezoelectric response. Science 357, 306–309. 10.1126/science.aai8535 28729511

[B46] Yu W. Li F. Yu L. Niazi M. R. Zou Y. Corzo D.. (2018). Single crystal hybrid perovskite field-effect transistors. Nat. Commun. 9:5354. 10.1038/s41467-018-07706-9 30559392 PMC6297354

[B47] Zhang J. Han S. Liu X. Wu Z. Ji C. Sun Z.. (2018). A lead-free perovskite-like hybrid with above-room-temperature switching of quadratic nonlinear optical properties. Chem. Commun. 54, 5614–5617. 10.1039/C8CC02496K 29770371

[B48] Zhang Y. Liao W. Q. Fu D. W. Ye H. Y. Liu C. M. Chen Z. N.. (2015). The first organic-inorganic hybrid luminescent multiferroic: (Pyrrolidinium)MnBr_3_. Adv. Mater. 27, 3942–3946. 10.1002/adma.201501026 26011784

[B49] Zhou L. Liao J. F. Huang Z. G. Wei J. H. Wang X. D. Chen H. Y.. (2019). Intrinsic self-trapped emission in 0D lead-free (C4 H14 N2)2 In2 Br10 single crystal. Angew. Chem. Int. Ed. Engl. 58, 15435–15440. 10.1002/anie.201907503 31448499

[B50] Zhu H. Fu Y. Meng F. Wu X. Gong Z. Ding Q.. (2015). Lead halide perovskite nanowire lasers with low lasing thresholds and high quality factors. Nat Mater. 14, 636–642. 10.1038/nmat4271 25849532

[B51] Zhu H. Liu A. Luque H. L. Sun H. Ji D. Noh Y. Y. (2019). Perovskite and conjugated polymer wrapped semiconducting carbon nanotube hybrid films for high-performance transistors and phototransistors. ACS Nano. 13, 3971–3981. 10.1021/acsnano.8b07567 30844243

[B52] Zhumekenov A. A. Burlakov V. M. Saidaminov M. I. Alofi A. Haque M. A. Turedi B. (2017). The role of surface tension in the crystallization of metal halide perovskites. ACS Energy Lett. 2, 1782–1788. 10.1021/acsenergylett.7b00468

