# Supplementary material for: (1-C5H14N2Br)2MnBr4: A Lead-Free Zero-Dimensional Organic-Metal Halide With Intense Green Photoluminescence
Source: Front Chem. 2020 Apr 28;8:352. doi: 10.3389/fchem.2020.00352 (PMC7199156; doi:10.3389/fchem.2020.00352)
Supplement: Supplementary file 1 [file Presentation_1.pdf]

Supplementary Material

Table S1. Crystal data and structure refinement for $(1\text{-mPQBr})_2MnBr_4$ single crystal.

Empirical formula	$C_{10}H_{28}Br_6MnN_4$
Formula weight	738.76
Temperature/K	300.07
Crystal system	orthorhombic
Space group	$P2_12_12_1$
a/Å	8.272(6)
b/Å	15.982(10)
c/Å	17.489(11)
α/°	90
β/°	90
γ/°	90
Volume/Å^3	2312(3)
Z	4
ρ_{calc}g/cm^3	2.122
μ/mm^{-1}	10.940
F(000)	1404.0
Radiation	MoKα (λ = 0.71073)
2θ range for data collection/°	4.658 to 49.996
Reflections collected	28852

Independent reflections	4075 [R_{int}= 0.1482, R_{sigma}= 0.0915]
Data/restraints/parameters	4075/43/193
Goodness-of-fit on F^2	0.968
Final R indexes [I>=2σ (I)]	R_1= 0.0460, wR_2= 0.0761
Final R indexes [all data]	R_1= 0.0851, wR_2= 0.0857
Largest diff. peak/hole / e $Å^{-3}$	0.86/-0.58

[a] CCDC Number: 1979443

Table S2. Fractional Atomic Coordinates ($\times 10^4$) and Equivalent Isotropic Displacement Parameters ($Å^2 \times 10^3$) for $(1\text{-mPQBr})_2MnBr_4$ single crystal. Ueq is defined as 1/3 of of the trace of the orthogonalised UIJ tensor.

**Atom**	***x***	***y***	***z***	**U(eq)**
Br1	6598.7(19)	6808.9(8)	1898.1(7)	46.7(4)
Br2	9599.2(19)	4998.5(8)	2644.4(7)	48.6(4)
Br5	7704(2)	2942.9(8)	4100.6(8)	49.8(4)
Br3	4678(2)	4507.0(8)	2346.4(8)	56.5(4)
Br6	929(2)	6246.8(8)	4773.7(7)	55.8(5)
Br4	6065(2)	6080.7(9)	4050.9(7)	63.7(5)
Mn1	6776(3)	5537.0(11)	2758.9(10)	39.0(5)
N1	3584(7)	3908(4)	5247(3)	40(3)
C4	2241(8)	4363(5)	5631(3)	41(4)
C3	624(7)	4124(5)	5293(3)	46(4)

N2	620(7)	4251(5)	4447(3)	39(3)
C1	3578(7)	4050(6)	4398(3)	45(4)
C2	1959(8)	3790(5)	4061(3)	42(4)
C5	5193(8)	4102(7)	5601(4)	68(5)
N3	476(9)	7770(4)	3631(4)	54(4)
N4	3641(8)	7573(5)	2967(5)	67(4)
C8	2288(10)	7002(4)	2751(4)	55(4)
C9	674(9)	7436(6)	2843(4)	51(4)
C6	1841(9)	8342(6)	3851(5)	54(4)
C7	3464(10)	7907(6)	3759(5)	65(5)
C10	-1147(9)	8167(7)	3748(7)	99(7)

Table S3. Bond lengths and bond angles for $(1\text{-mPQBr})_2MnBr_4$ single crystal.

**Atom**	**Atom**	**Length/Å**	**Atom**	**Atom**	**Atom**	**Angle/°**
Br1	Mn1	2.534(2)	Br2	Mn1	Br1	106.44(9)
Br2	Mn1	2.497(3)	Br2	Mn1	Br3	113.54(9)
Br3	Mn1	2.498(3)	Br3	Mn1	Br1	108.4(9)
Br4	Mn1	2.491(3)	Br4	Mn1	Br1	104.21(9)
N1	C4	1.4872(15)	Br4	Mn1	Br2	114.43(9)
N1	C1	1.5029(17)	Br4	Mn1	Br3	109.14(10)

N1	C5	1.4997(16)	C4	N1	C1	111.7(5)
C4	C3	1.5127(16)	C4	N1	C5	112.09(15)
C3	N2	1.4923(16)	C5	N1	C1	112.28(14)
N2	C2	1.4921(15)	N1	C4	C3	111.13(15)
C1	C2	1.5202(16)	N2	C3	C4	110.82(14)
N3	C9	1.4873(16)	C2	N2	C3	112.29(14)
N3	C6	1.5029(15)	N1	C1	C2	110.15(14)
N3	C10	1.4993(16)	N2	C2	C1	110.11(14)
N4	C8	1.4921(15)	C9	N3	C6	111.9(6)
N4	C7	1.4919(16)	C9	N3	C10	112.12(14)
C8	C9	1.5132(16)	C10	N3	C6	112.34(15)
C6	C7	1.5208(16)	C7	N4	C8	112.32(15)
			N4	C8	C9	110.79(15)
			N3	C9	C8	111.08(14)
			N3	C6	C7	111.0(6)
			N4	C7	C6	110.37(12)

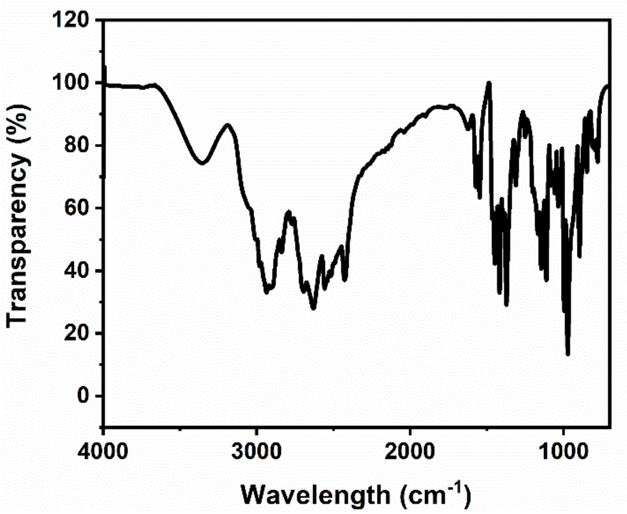

Figure S1. FTIR spectrum of (1-mPQBr)$_2$MnBr$_4$ single crystal.

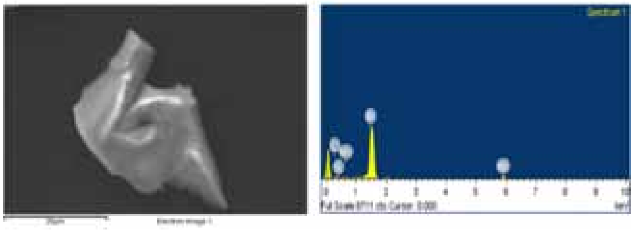

Figure S2. SEM-EDS spectra of (1-mPQBr)$_2$MnBr$_4$.

Table S4. SEM-EDS results of element components and ratio of (1-mPQBr)$_2$MnBr$_4$ single crystal.

Element	Weight/%	Atomic/%	Ratio
C K	18.05	57.55	10.56
Mn K	7.83	5.45	1
Br L	73.47	35.21	6.46

Table S5 Comparison of the photoluminescent data of title compound and the reported ones

Compound	Space group	Peak position (nm)	FWHM (nm)	PLQY (%)	Average Lifetime
$(C_9NH_{20})_2MnBr_4$[1]	C2/c	528	64	81.08	326 μs
$[Bu_4N]_2[MnBr_4]$[2]	$P2_12_12_1$	520	N/A	47	350 μs
$[Ph_4P]_2[MnBr_4]$[2]	C2/c	524	N/A	47	350 μs
$C_{10}H_{12}N_2MnBr_4$[3]	P-1	523	N/A	N/A	213 μs
$(Bz(Me)_3N)_2MnBr_4$[4]	$P2_1$	520	53.5	68	340 μs
$(Diisopropylammonium)_2MnBr_4$[5]	Iba2	525	62.9	62.2	1.44 ns
$[pyrrolidinium]_2MnBr_4$[6]	*P*21/*c*	525	N/A	51.41	N/A
$(C_9NH_{20})_9[Pb_3Br_{11}](MnBr_4)_2$[7]	P31c	528	67	49.8	114 μs
This work	$P2_12_12_1$	520	43	60.70	183 μs

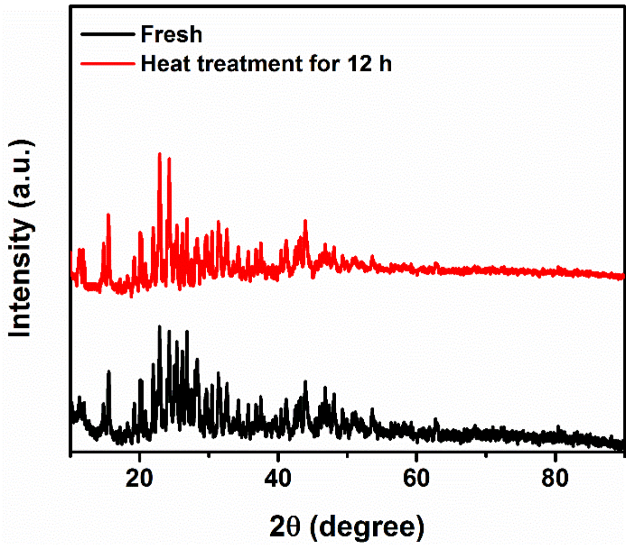

Figure S3. PXRD patterns of (1-mPQBr)$_2$MnBr$_4$ powders before and after heat treatment at 150 °C for 12 h.

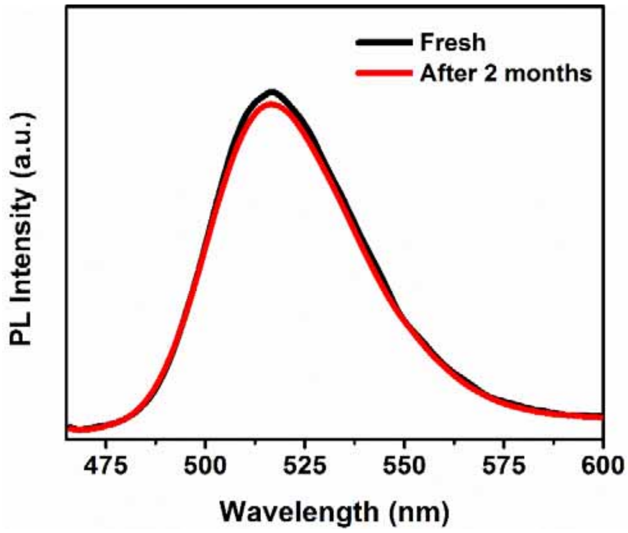

Figure S4. Photoluminescence stability of (1-mPQBr)$_2$MnBr$_4$ single crystals after exposure to ambient conditions for two months.

Reference

1. Li, M.; Zhou, J.; Molokeev, M. S.; Jiang, X.; Lin, Z.; Zhao, J.; Xia, Z., Lead-Free Hybrid Metal Halides with a Green-Emissive [MnBr4] Unit as a Selective Turn-On Fluorescent Sensor for Acetone. Inorg. Chem. 2019, 58 (19), 13464-13470.

2. Gong, L. K.; Hu, Q. Q.; Huang, F. Q.; Zhang, Z. Z.; Shen, N. N.; Hu, B.; Song, Y.; Wang, Z. P.; Du, K. Z.; Huang, X. Y., Efficient modulation of photoluminescence by hydrogen bonding

interactions between inorganic [MnBr4](2-) anions and organic cations. Chem. Commun. 2019, 55 (51), 7303-7306.

3. Jana, A.; Zhumagali, S.; Ba, Q.; Nissimagoudar, A. S.; Kim, K. S., Direct emission from quartet excited states triggered by upconversion phenomena in solid-phase synthesized fluorescent lead-free organic–inorganic hybrid compounds. J. Mater. Chem. A 2019, 7 (46), 26504-26512.

4. Bai, X. W.; Zhong, H. Z.; Chen, B. K.; Chen, C.; Han, J. B.; Zeng, R. S.; Zou, B. S., Pyridine-Modulated Mn Ion Emission Properties of C10H12N2MnBr4 and C5H6NMnBr3 Single Crystals. J. Phys. Chem. C 2018, 122 (5), 3130-3137.

5. Morad, V.; Cherniukh, I.; Pöttschacher, L.; Shynkarenko, Y.; Yakunin, S.; Kovalenko, M. V., Manganese(II) in Tetrahedral Halide Environment: Factors Governing Bright Green Luminescence. Chem. Mater. 2019, 31 (24), 10161-10169.

6. Jiang, C.; Zhong, N.; Luo, C.; Lin, H.; Zhang, Y.; Peng, H.; Duan, C. G., (Diisopropylammonium)2MnBr4: a multifunctional ferroelectric with efficient green-emission and excellent gas sensing properties. Chem. Commun. 2017, 53 (44), 5954-5957.

7. Wu, Y. -X.; Wang, C. -F.; Li, H. -H; Jiang, F.; Shi, C.; Ye, H. -Y.; Zhang, Y., Highly Efficient and Uncommon Photoluminescence Behavior Combined with Multiple Dielectric Response in Manganese(II) Based Hybrid Phase Transition Compounds. Eur. J. Inorg. Chem. 2020, 4, 394-399.
